# Performance Evaluation of Analog Beamforming with Hardware Impairments for mmW Massive MIMO Communication in an Urban Scenario

**DOI:** 10.3390/s16101555

**Published:** 2016-09-22

**Authors:** Sonia Gimenez, Sandra Roger, Paolo Baracca, David Martín-Sacristán, Jose F. Monserrat, Volker Braun, Hardy Halbauer

**Affiliations:** 1 iTEAM Research Institute, Universitat Politècnica de València, Valencia 46022, Spain; sogico@iteam.upv.es (S.G.); damargan@iteam.upv.es (D.M.-S.); jomondel@iteam.upv.es (J.F.M.); 2 Nokia Bell Labs, Stuttgart 70435, Germany; paolo.baracca@nokia-bell-labs.com (P.B.); volker.braun@nokia-bell-labs.com (V.B.); hardy.halbauer@nokia-bell-labs.com (H.H.)

**Keywords:** massive MIMO, analog beamforming, hardware impairments, millimeter-wave

## Abstract

The use of massive multiple-input multiple-output (MIMO) techniques for communication at millimeter-Wave (mmW) frequency bands has become a key enabler to meet the data rate demands of the upcoming fifth generation (5G) cellular systems. In particular, analog and hybrid beamforming solutions are receiving increasing attention as less expensive and more power efficient alternatives to fully digital precoding schemes. Despite their proven good performance in simple setups, their suitability for realistic cellular systems with many interfering base stations and users is still unclear. Furthermore, the performance of massive MIMO beamforming and precoding methods are in practice also affected by practical limitations and hardware constraints. In this sense, this paper assesses the performance of digital precoding and analog beamforming in an urban cellular system with an accurate mmW channel model under both ideal and realistic assumptions. The results show that analog beamforming can reach the performance of fully digital maximum ratio transmission under line of sight conditions and with a sufficient number of parallel radio-frequency (RF) chains, especially when the practical limitations of outdated channel information and per antenna power constraints are considered. This work also shows the impact of the phase shifter errors and combiner losses introduced by real phase shifter and combiner implementations over analog beamforming, where the former ones have minor impact on the performance, while the latter ones determine the optimum number of RF chains to be used in practice.

## 1. Introduction

Nowadays, one of the most active research areas in the wireless communication field is the design of communication techniques for fifth generation (5G) cellular systems. Although the final technical components of the 5G system are still under discussion, important progress has already been made towards the definition of the set of scenarios and requirements to be addressed by these systems. In particular, the European project Mobile Enablers for the Twenty-Twenty Information Society (METIS) played a key role in this direction by stating that, among other goals, the 5G system shall boost the user data rate of 4G cellular systems by a factor ranging from 10 to 100 [1].

A promising technology to meet this specific 5G requirement is the use of advanced multiple-input multiple-output (MIMO) techniques with large antenna arrays, where the number of antenna elements is much higher than the number of users to be served, also known as massive MIMO [2]. Massive MIMO techniques imply the use of, at least, an order of magnitude more antenna elements than in current systems [3], and their application can be twofold: either it can be used to compensate the propagation losses of backhaul and access links through beamforming, or it can be used to boost capacity with multi-stream transmission. In fact, although it has been historically associated with beamforming, current trends point towards the application of MIMO spatial multiplexing techniques in conjunction with beamforming to best exploit the richness of the channel [4].

Unfortunately, one of the showstoppers for the successful incorporation of massive MIMO into cellular networks is the large physical size of the antenna arrays at currently used cellular frequencies. For this reason, massive MIMO is being considered in conjunction with millimeter-Wave (mmW) frequencies [3,5], where antenna arrays of reasonable physical sizes are feasible [6]. From a signal processing perspective, transmission with massive arrays increases the processing complexity derived from the computation of the required precoders when digital schemes are applied to hundreds of antennas. Multi-antenna digital precoding is carried out at baseband and, thus, it requires an architecture with as many radio-frequency (RF) chains as antenna ports to do the digital to analog data conversion and subsequent upmixing to RF. However, the use of one RF chain per antenna is not only a very costly option for massive MIMO systems, but it also leads to a extremely high power consumption [3,7]. Motivated by this, there is now a growing interest in alternative transmission schemes based on analog beamforming (ABF) and hybrid beamforming (HBF) architectures, where all or part of the processing is based on radio-frequency beamforming. Many ABF and HBF approaches have been proposed in the last years, both for single-user (SU) [8,9] and multi-user (MU) transmissions [10,11], all of them using a substantially reduced number of RF chains with respect to the number of antennas. The potential of these schemes is well-known; however, to the best of the authors’ knowledge, there is not yet a clear view on the performance of the different beamforming alternatives in a complete system with multiple transmission and reception points and a realistic channel model. In this direction, some preliminary tests were carried out for a single-link case in [12] and for a multi-link case in [13].

System level simulations usually rely on a set of ideal assumptions that may have a non negligible impact on the accuracy of the assessments based on these simulations [13]. One such assumption is the perfect knowledge of the channel state at the transmitter, the receiver or both entities. In practice, channel measurements present errors and, additionally, channel state changes within the time elapsed from the channel measurement until the transmission/reception phase. Therefore, perfect channel knowledge is not realistic. Another common assumption is the use of ideal hardware components in all the devices involved in the communication. However, the presence of non-ideal devices in real implementations brings in several error sources that may affect the performance of the algorithms under study. In particular, real phase shifter implementations introduce phase errors whose magnitude was studied in [14,15], and non-ideal combiners introduce power losses that were characterized, for instance, in [16]. The effect of hardware-constrained base stations in massive MIMO systems was extensively analyzed in [17], where a scaling law of the increase of some particular imperfections with the number of antennas was provided. In addition, hardware constraints impose practical limitations that should be desirably considered in the algorithms design. This was highlighted in [18,19], which propose constant envelope precoders suitable for the use of power-efficient RF power amplifiers as a constraint to avoid the problems of such non-ideal amplifiers with their inherent non-linear behaviour. Overall, it is of paramount importance to consider the effect of hardware impairments and other practical limitations when analyzing the system performance achieved by massive MIMO cellular systems.

Motivated by the lack of concluding results on the mmW multi-cell performance of ABF, this paper provides a thorough assessment of ABF schemes for MU single-stream transmission under realistic channel conditions in the lower edge of the mmW frequency band. In particular, a carrier frequency of 28 GHz is considered, which, although it does not strictly belong to the mmW band (defined from 30 and 300 GHz), it has been widely adopted by industry as a mmW frequency, as any other frequency above 10 GHz [20]. To this end, exhaustive system simulations have been conducted, assuming a multi-cell deployment in which the channel is accurately emulated and there is a coexistence of users with very different channel conditions. The main objective is to complement existing theoretical studies by assessing under which conditions ABF schemes can approach the performance of fully-digital precoders. In addition, the performance of the analog schemes with inter-beam interference in the system (i.e., when multiple users are simultaneously scheduled) is also investigated.

Simulation results with ideal assumptions on aspects such as the timely availability of channel state information (CSI), ideal phase-shifters and combiners for ABF and lack of per-antenna power constraints (PAPC) will be contrasted with results including more realistic assumptions on these aspects. More specifically, the following realistic assumptions were selected and analyzed:

- outdated CSI at the transmitters;
- PAPC in the precoding design;
- Inaccuracy errors caused by real phase shifters;
- Losses introduced by real combiners.

The rest of the paper is organized as follows. Section 2 details the system model and multi-antenna schemes assessed in this work, namely fully-digital and analog schemes. Section 3 describes the simulation assumptions and includes the performance evaluation with ideal assumptions. Section 4 and Section 5 are devoted to the performance evaluation including practical limitations and hardware impairments, respectively. Finally, Section 6 draws the main findings of the paper.

## 2. System Model and Evaluated Precoding Schemes

This paper studies an MU mmW downlink system with $N_{BS}$ Base Stations (BSs) and *K* User Equipment devices (UEs). Each BS is equipped with $N_{t}$ antennas and $P\leq N_{t}$ RF chains. For the sake of simplicity in the simulations, a single narrow receive beam is assumed at each UE (which would require an antenna array in practice), in order to simulate a beamforming gain at the receiver as in [21]. Furthermore, the pointing direction of the directive beam is selected by the user to maximize the signal to noise ratio (SNR) at the receiver.

The downlink transmission resources are partitioned in the time-frequency plane. In the time domain, the resource space is divided into subframes of a certain time duration. In the frequency domain, resources are grouped in frequency blocks (FB). The minimum time-frequency resource is known as resource block (RB), and it is composed of one frequency block during one subframe period. This terminology is the same as the one used by the the 3rd Generation Partnership Project (3GPP) and the METIS project [1] to carry out performance evaluations.

In order to exploit the BS multi-antenna architecture to either multiplex different data streams or to provide beamforming gains to the different users, some form of precoding or beamforming must be applied at the transmitter side. The schemes considered in this work are next detailed.

### 2.1. Fully Digital Precoding Schemes

In fully digital implementations, where $P=N_{t}$, precoding is commonly done in the digital baseband, thus allowing a very flexible design of the precoding matrix or vector. Many fully-digital precoding designs for MU-MIMO systems can be found in the literature [22]. Although the optimum precoders, in terms of achieving capacity, require non-linear implementation, in this work, we will focus on linear precoding, which is known to provide a good tradeoff between performance and complexity. In particular, we selected the well-known Maximum Ratio Transmission (MRT) linear precoder, which is designed to maximize the SNR at the intended user and, thus, it only requires the knowledge of the channel vector between UE *k* and its serving BS in FB *b*, denoted by $h_{k,b}\in C^{1\times N_{t}}$. The MRT precoder in FB *b* for UE *k*, $w_{{BB}_{k,b}}$, is computed as:

(1) $$w_{{BB}_{k,b}}=\frac{h_{k,b}^{H}}{\left|\right|h_{k,b}\left|\right|},$$

where the superscript ${}^{H}$ stands for conjugate-transpose operation. The main reason for the selection of this digital precoder is that, when the number of antennas is very high, its performance is very close to the performance of other precoding schemes that require higher computational complexity, such as zero-forcing (ZF) or regularized ZF [23].

We focus on digital MU transmission, that is, a set of as many users as BS antennas can be served simultaneously at each subframe by multiplexing them in the spatial domain. Furthermore, we will investigate the effect of multiplexing different sets of users in different FBs of each subframe. To this end, two variants of digital precoding have been implemented and simulated, denoted by digital precoding non frequency selective (DP NFS) and digital precoding frequency selective (DP FS). In DP NFS, all FBs are allocated to a single set of UEs per subframe, while, in DP FS, the allocation is optimized per RB, thereby allowing different sets of UEs to be scheduled per subframe in different FBs.

### 2.2. Analog Beamforming Schemes

As mentioned above, in mmW systems, the high number of antennas required to achieve a high quality link makes fully-digital implementations hardly feasible in practice, due to the high cost and power consumption of having as many RF chains as antennas. For that reason, less complex precoding schemes either in the analog (i.e., radio-frequency) domain or combining the processing in both digital baseband and analog domain are receiving increasing attention [11].

**Figure 1:**
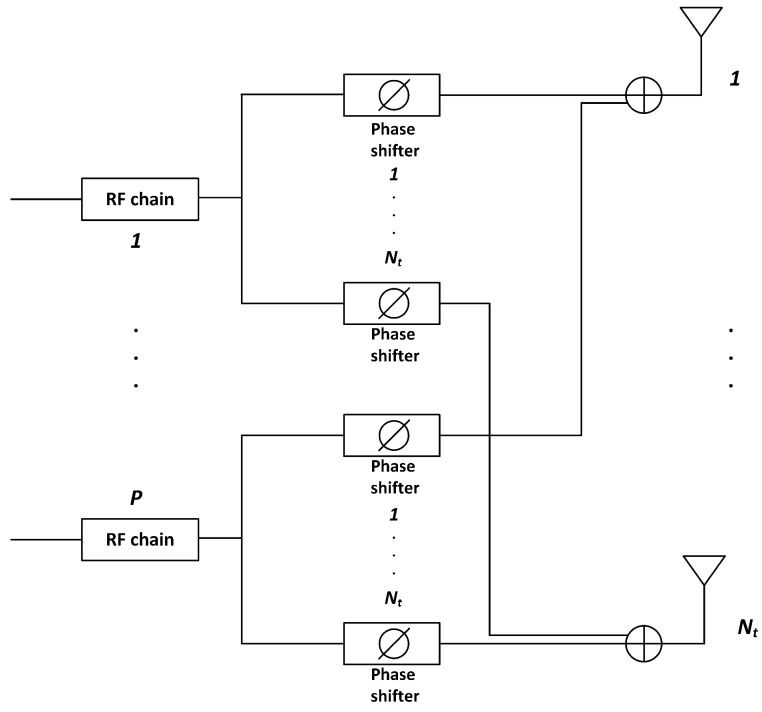
Transceiver considered at the Base Station (BS), which is equipped with $N_{t}$ antenna elements and *P* radio-frequency (RF) chains.

In RF beamforming, phase shifts are applied to each antenna element of the array in order to steer the beam towards a certain direction. Figure 1 shows an example of analog beamforming (ABF) architecture, with $P<<N_{t}$ RF chains and a set of $N_{t}$ phase shifters applied to each RF chain. The outputs of the *i*-th phase shifters of every RF chain are combined to feed the *i*-th antenna element in the array. Using this architecture, *P* data streams can be simultaneously transmitted with full array gain and up to *P* different users can be spatially multiplexed. In this work, we consider a simple and practical method for mmW beamforming. Motivated by the fact that the channel at high frequencies is characterized by limited scattering, in particular for the UEs in line of sight (LoS) condition, we assume that (a) beamformers are designed only in the RF domain, and (b) the digital stage is optimized to schedule up to *P* UEs in each subframe, each UE being served by a specific RF beamformer. More sophisticated schemes could be used [10,11], for instance, by optimizing the digital precoding weights to be used in the baseband domain. However, these schemes are expected to provide relevant gain only when most of the UEs have a highly frequency selective channel, as in scenarios where there are mostly UEs in non line of sight (NLoS) conditions. Therefore, in our analysis, we will intentionally compare a simple ABF scheme against fully DP. Future work will include a more thorough evaluation on how much of the performance gap between these two schemes can be filled by more advanced (but at the same time more complex) hybrid beamformers.

The RF beamforming vector for the *k*-th UE, denoted by $w_{RF_{k}}$, is selected from a codebook based on the discrete Fourier transform (DFT) matrix, which provides a grid of beams such as in [10], useful also for channel limited feedback setups. Elements of the codebook are of this form:

(2) $$w_{RF}^{\left(c\right)}=\frac{1}{\sqrt{N_{t}}}{\left[1,e^{\frac{j2\pi c}{C}},\ldots ,e^{\frac{j2\pi c}{C}\left(N_{t}-1\right)}\right]}^{T},$$

where c=0,1,…,C−1 is the index of the codebook elements and *C* the number of vectors in the codebook. We assume $C=N_{t}$, that is to say, maximum diversity order. The specific beamforming vector for UE *k* is chosen to maximize the sum rate over the whole allocated band B as:

(3) $$w_{{RF}_{k}}=\underset{w_{RF}^{\left(c\right)}}{arg max}\sum_{b\in B}\log_{2}\left(1+\frac{P_{t}{\left|h_{k,b}w_{RF}^{\left(c\right)}\right|}^{2}}{N+I}\right),$$

where $P_{t}$ is the transmit power, and *N* and *I* are the noise and long term interference power at the receiver, respectively. Although many other codebook designs could be used, DFT-based precoding is chosen in this work to achieve a good tradeoff between complexity and performance.

Note that the whole bandwidth is available at each RF chain and multiple UEs are multiplexed in the spatial domain. Equal power sharing among users is considered, meaning, for example, that, when *P* users are simultaneously multiplexed by a BS, the available power per RF chain must be scaled by 1/P to fulfill a per-BS power restriction.

## 3. Simulation Setup and Results under Ideal Assumptions

### 3.1. Channel Modeling and Deployment Considerations

We consider the 3D mmW channel model proposed in [24], characterizing an urban micro-cellular scenario. This model is based on real measurements taken in New York City. It is consistent with the 3GPP ray-based modeling methodology, and includes characterization of the channel in azimuth, elevation and polarization. This model takes into account channel variability in the frequency and time domains, considering also the actual correlation between antennas depending on the geometry of the antenna array deployment. The propagation condition has been taken from the urban micro 3GPP channel model [25], which classifies users in LoS or NLoS condition by means of a probability distribution function of the distance between BS and UE. Simulations are performed in the lower edge of the mmW band, using a carrier frequency of 28 GHz and a system bandwidth of 1 GHz. Unless otherwise stated, perfect channel knowledge is assumed at the transmitter and receiver side.

**Figure 2:**
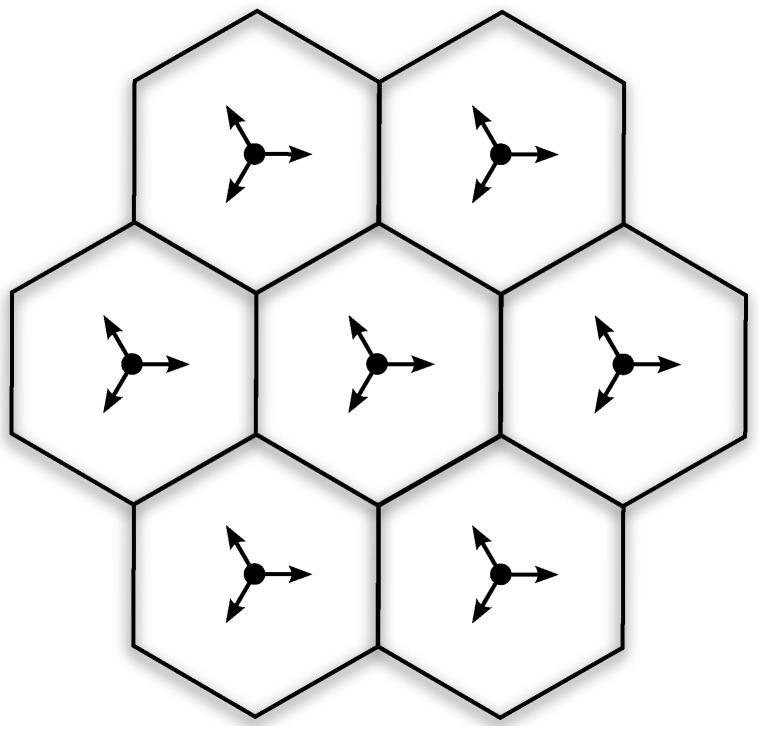
Seven-site layout considered for the simulations. Each site covers three $120^{\circ }$ sectors, each one equipped with the antenna array boresight indicated by the arrows.

The simulation setup considers a wrap-around configuration of the seven-site layout depicted in Figure 2. The sites are deployed in an urban scenario with inter site distance of 200 m, which entails having approximately 70% of UEs in LoS condition. Each site includes three $120^{\circ }$ sectors (cells), each one served by a BS equipped with a horizontal uniform linear array of 64 antenna elements. The antenna pattern of each element is parabolic as in [25], and then defined by a maximum gain (8 dBi), front-to-back ratio (20 dB), and half-power beamwidth ($70^{\circ }$). Fifty UEs are randomly deployed per site, leading to an average of 16.7 UEs per BS. As already said, the modeling of beamforming at the UE side is simplified. Specifically, we assume that the UE is equipped with an array of antenna elements whose resulting pattern is parabolic with a maximum gain of 12 dBi, a front-to-back ratio of 20 dB, and a half-power beamwidth of $45^{\circ }$ (the latter calculated according to [21]). In addition, we consider that the UE beamformer is always pointing to the direction that maximizes the SNR at the receiver. For all the simulated schemes, we assume a per-site power restriction of 40 dBm, with equal power allocated to each sector. In addition, equal power allocation among the active RF chains is considered at each BS for the analog implementations. Other important simulation parameters are listed in Table 1.

### 3.2. Performance with Ideal Assumptions

In this section, a performance comparison among digital precoding and analog beamforming is carried out under ideal assumptions. More specifically, we will assume perfect and timely CSI available at the transmitters, ideal phase-shifters and combiners in the analog scheme and lack of any per-antenna power constraint in digital precoding. Recall that the maximum number of simultaneously scheduled users per BS is limited by the number of available RF chains *P* in the analog beamforming scheme, and by the number of BS antennas in the digital precoding schemes. Moreover, ABF and DP NFS schemes keep the same set of scheduled UEs along the whole band, whereas DP FS can schedule a different set of users per FB.

The average number of different UEs simultaneously allocated per subframe is reported in Table 2. It can be observed how the analog scheme makes the most of the number of parallel RF chains until reaching a certain number of chains, from which no more users are multiplexed. In particular, for the simulation setup in this work, even with 16 RF chains, the analog scheme only multiplexes 11.9 users on average. Regarding the DP schemes, they show a higher average number of multiplexed users per subframe than the ABF ones. It is worth noting also that the maximum number of UEs per BS is 16.7 and the DP FS scheme can meaningfully reach this number by performing a more elaborated per-FB allocation.

**Figure 3:**
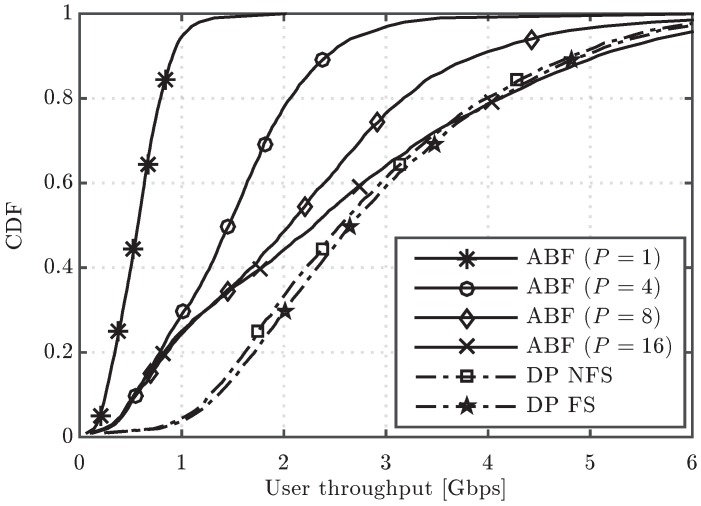
Cumulative distribution function (CDF) of user throughput for the analog beamforming (ABF) and digital precoding (DP) evaluated schemes.

**Figure 4:**
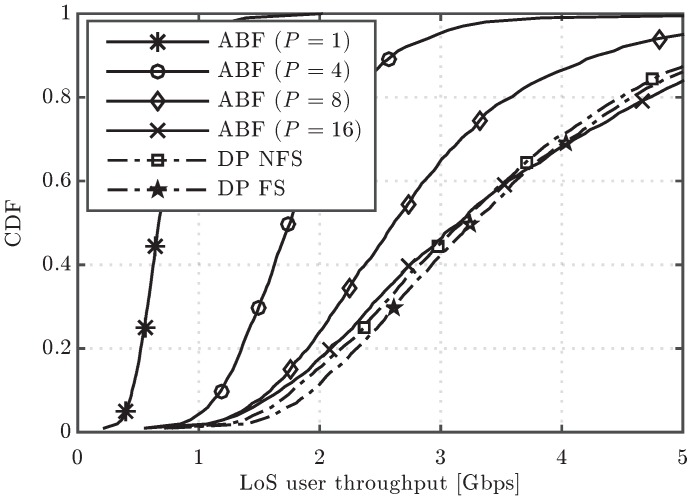
CDF of user throughput for the ABF and DP evaluated schemes considering only line of sight (LoS) users.

**Figure 5:**
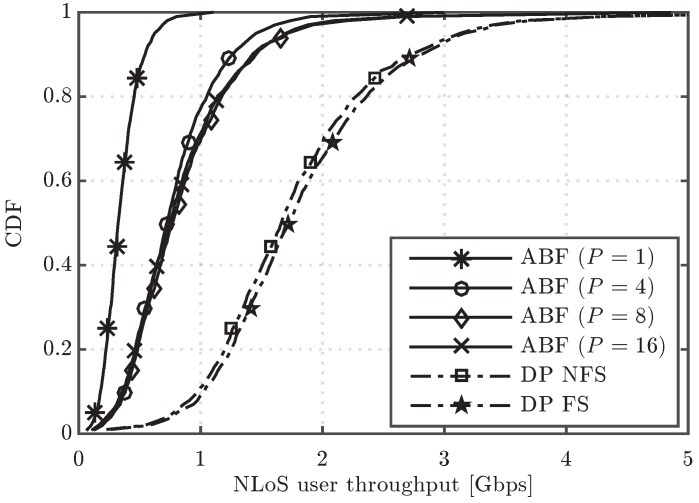
CDF of user throughput for the ABF and DP evaluated schemes considering only non line of sight (NLoS) users.

Figure 3 shows the cumulative distribution function (CDF) of the user throughput for the algorithms under study. It is observed that, in the upper part of the CDF curves, the performance of ABF approaches the one of DP when the number of RF chains increases, but a significant gap in performance appears in the lower part, even when the number of RF chains is increased from four to sixteen. For the sake of completeness, the system performance having users in different conditions can be observed in Figure 4 and Figure 5, which show the CDF curves of user throughput for only those users in LoS and NLoS, respectively. It can be seen that increasing the number of RF chains *P* in the analog scheme has a different impact depending on whether the user is in LoS or NLoS. Note that no benefit for NLoS users is obtained when increasing *P* beyond eight, since the greater the number of simultaneously scheduled users, the higher the inter-beam interference generated into the system. On the other hand, for the users in LoS, less affected by the interference, increasing *P* improves the user throughput, which reaches similar values to the ones obtained by the digital precoding schemes with sufficiently large *P*. Concerning the two considered DP variants, both schemes perform very similarly in the three cases (all the UEs, only UEs in LoS and only UEs in NLoS), but the DP FS attains slightly superior performance when compared to DP NFS, due to its higher flexibility in scheduling different sets of UEs in different RBs.

Finally, average user throughput, 5th percentile of the user throughput and average cell throughput values are compared in Table 3 for all the simulated algorithms. Table 3 also shows the gains with respect to a scheme without spatial multiplexing (i.e., ABF with P=1), considered here as the baseline. This comparison shows the significant benefit of spatially multiplexing multiple users in each subframe. Focusing, for example, on the case of the ABF with four RF chains, we observe that the gain in the average user throughput is about 150% when compared to the baseline scheme with only one RF chain. Results also highlight the main limitation of the ABF schemes, which, while able to approach the performance of the DP in terms of average user and cell throughput, cannot provide large gains for the 5th percentile user throughput, even when the number of RF chains *P* increases.

## 4. Performance with Practical Limitations

### 4.1. Performance with Outdated Channel State Information

A very usual ideal assumption for simulation is the perfect knowledge of the instantaneous (per-subframe) channel at every BS, which allows the BS precoder to be adapted to the actual channel. However, practical systems must often deal with the problem of having imperfect CSI. In frequency division duplex (FDD) systems, the downlink channel is estimated at the UE and fed back to the BS. These estimation and feedback stages can, in practice, impair the CSI with quantization noise, due to the usually limited resources on the feedback channel. In addition, the presence of noise in the channel during the transmission of training sequences for CSI estimation contributes to an imperfect channel estimation. The latter effect is also present in the channel estimation stage of time division duplex (TDD) systems. Another important practical limitation in both FDD and TDD systems is the unavoidable delay between the instant the channel is estimated and the instant the channel is used in designing the precoder for data transmission, which, in turn, can further degrade the system performance.

In this work, we will focus on the effect of outdated CSI over the performance of ABF with P=4, DP NFS and DP FS. To this end, it is here assumed that each BS receives an update of the channel coefficients only every certain period *T*, higher than the subframe duration $T_{s}=1$ ms. It is worth noting that we consider *T* values that cause changes only in the small scale fading components of the channel, i.e., the large scale fading components like path loss, shadowing and path angles of arrival/departure remain constant during *T*. For a better illustration of the small scale channel variation during the period *T*, the results will be presented as a function of the product $f_{D}\Delta T$, where $f_{D}=vf/c$ is the maximum Doppler shift at carrier frequency *f* with user speed equal to *v*, and $\Delta T=T-T_{s}$ is the CSI delay with respect to the subframe duration.

**Figure 6:**
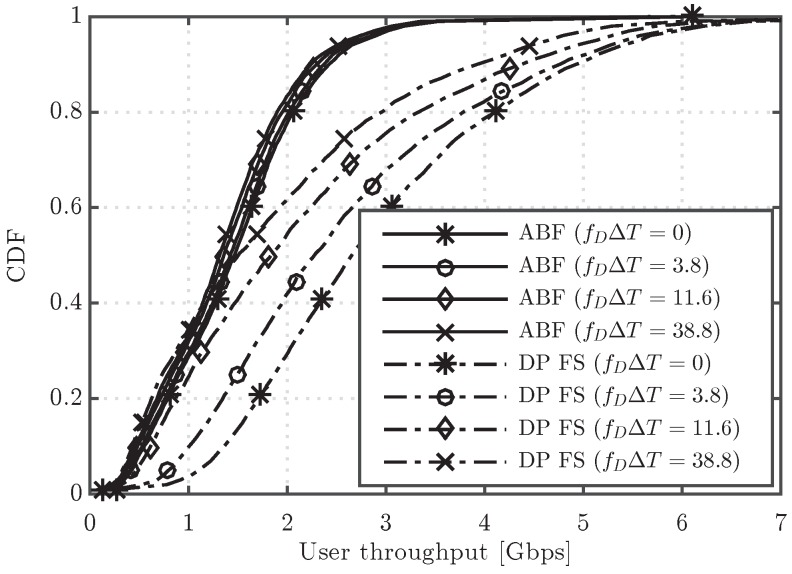
CDF of user throughput for the ABF with P=4 and digital precoding frequency selective (DP FS) schemes considering different values of $f_{D}\Delta T$.

**Figure 7:**
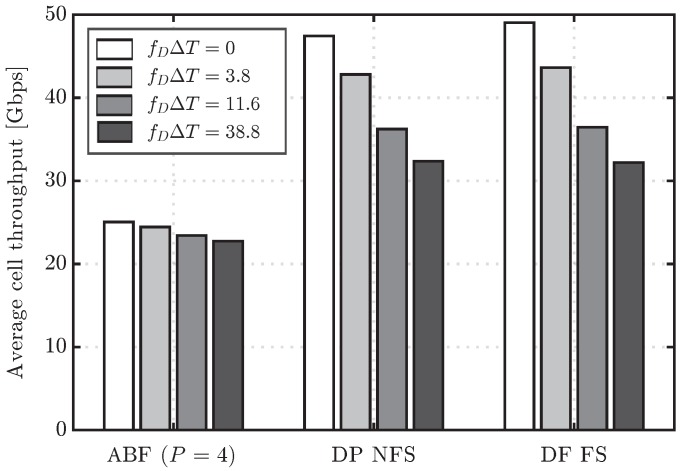
Average cell throughput for the ABF with P=4 and DP evaluated schemes considering different values of $f_{D}\Delta T$.

Figure 6 shows the CDF of user throughput for the ABF and DP FS schemes, considering different values of the channel update period, in particular T=1,50,150 and 500 ms, which lead to $f_{D}\Delta T=0,\;3.8,\;11.6$ and 38.8, respectively. Note that $f_{D}\Delta T=0$ corresponds to the ideal case, where the channel is supposed to be updated every subframe. It can be first observed that the ABF scheme is more robust to outdated CSI, as it presents a nearly negligible performance degradation with $f_{D}\Delta T$. Such degradation of ABF is also much lower than for the DP FS approach, which results in a reduced performance gap between ABF and DP as *T* increases. The lower robustness to outdated CSI of DP is well justified by the fact that this scheme has a strong dependence on the small scale channel fading, whereas the ABF mainly relies on the large scale components, since it typically tends to point the beamformer towards the angle of departure of the strongest path. In fact, while in a practical mmW system it is expected that the channel is tracked every few milliseconds or few tens of milliseconds, the higher values of *T* considered here, e.g., T=150,500 ms, are useful to understand the robustness of the different schemes when there is basically no knowledge of the small scale fading, and, thus, beamformers are designed mainly based on the large scale fading components of the channel. In such a scenario, ABF, by simply selecting the beam that points toward the direction that maximizes the signal to interference plus noise ratio (SINR) at the receiver, turns out to be much more robust than DP. For completeness, the average cell throughput for different values of the channel update period including also the DP NFS scheme is depicted in Figure 7. The latter results also confirm the robustness of ABF to the effect of outdated CSI, which presents a maximum penalty in cell throughput of 9.8% for $f_{D}\Delta T=38.8$. On the other hand, the cell throughput values of DP FS and DP NFS are reduced up to 32% and 34%, respectively. Regarding the comparison between DP NFS and DF FS, both show very similar performance, meaning that the additional complexity of DP FS to adapt the precoder per RB does not pay off with an outdated CSI.

### 4.2. Performance with PAPC

In practical multi-antenna systems, each element of the antenna array may be powered by its own amplifier and, thus, is limited by the linearity of that amplifier. For that reason, precoding schemes should apply a more restrictive assumption than the sum power constraint, that is, a per-antenna power constraint. In order to study the effect of limiting the maximum available power per antenna in the DP NFS and DP FS schemes, we consider, in this section, a second digital precoding alternative that takes into account this practical limitation: the Equal-Gain Transmission (EGT) scheme with per-antenna power constraints [19]. EGT ensures the allocation of the same amount of power per element of the antenna array. Moreover, its implementation is simple and has shown a bounded performance degradation in comparison to the MRT precoder when the number of antenna elements in the array is sufficiently high [26]. If the channel vector between UE *k* and its serving BS in FB *b* is expressed as:

(4) $$h_{k,b}={\left[|h_{1}|e^{j\theta_{1}},|h_{2}|e^{j\theta_{2}},\ldots ,\left|h_{N_{t}}\right|e^{j\theta_{N_{t}}}\right]}^{T},$$

then, the corresponding EGT precoding vector for UE *k* in FB *b*, $w_{{EGT}_{k,b}}$, is

(5) $$w_{{EGT}_{k,b}}=\frac{1}{\sqrt{N_{t}}}{\left[e^{-j\theta_{1}},e^{-j\theta_{2}},\ldots ,e^{-j\theta_{N_{t}}}\right]}^{T}.$$

As before, equal power allocation among users is considered. Note that PAPC is implicitly satisfied by the ABF scheme considered in this work, and, thus, the selection of EGT for DP allows a fair comparison from a power allocation perspective.

**Figure 8:**
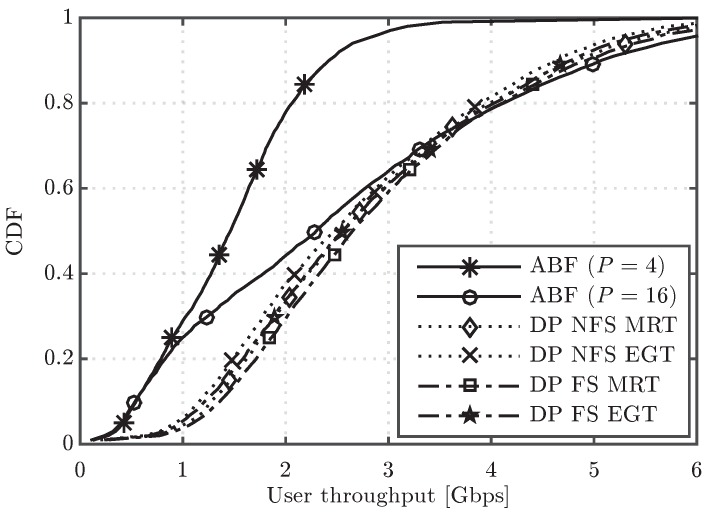
CDF of user throughput for the ABF, Maximum Ratio Transmission (MRT) and Equal-Gain Transmission (EGT) evaluated schemes.

**Figure 9:**
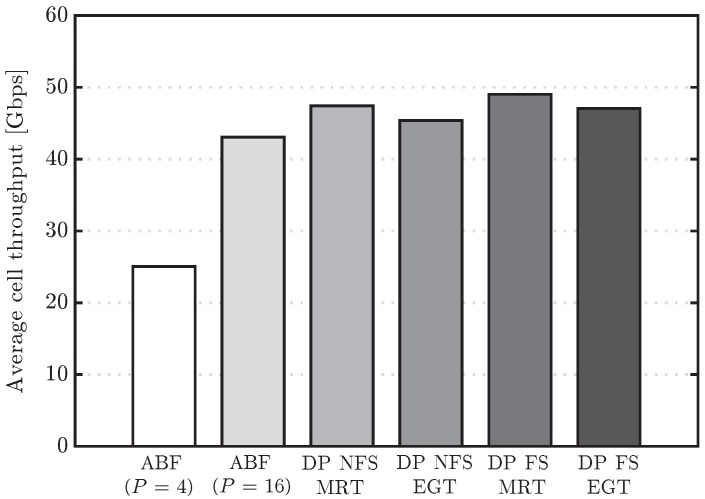
Average cell throughput values for the ABF, MRT and EGT evaluated schemes.

Figure 8 shows the CDF of user throughput for the two digital precoding alternatives, MRT and EGT with PAPC, both of them configured according to the two scheduling variants under study (DP NFS and DP FS). The performance results of ABF with P=4 and P=16 RF chains with ideal assumptions are also included in the figure for the sake of comparison. In general, the EGT precoder worsens the throughput performance of both DP variants, although not very significantly. A meaningful result is that MRT DP NFS achieves very similar performance to EGT DP FS. Therefore, the performance loss due to the PAPC constraint can be, in this case, compensated by a more elaborated scheduler (per FB instead of per subframe). Moreover, the power per antenna element limitation brings the performance of digital precoders a bit closer to the one of ABF with P=16 RF chains, i.e., with the same multiplexing capacity, although there is still a substantial performance gap for NLoS UEs (lower part of the CDF curve). Finally, the average cell throughput values for the ABF, MRT and EGT evaluated schemes are included in Figure 9. From this figure, we can confirm that the EGT-PAPC precoder causes approximately a 4% performance loss over both DP variants, a percentage much lower than the one caused by outdated CSI over DP.

## 5. Performance with Hardware Impairments

### 5.1. Performance with Phase-Shifter Errors

As shown in Figure 1, in RF beamforming, phase shifts are applied to each antenna element of the array in order to steer the beam towards a certain direction. In real implementations, the conformed beam is altered due to the phase errors introduced by the non-ideal phase shifters. In order to study the impact of this effect, the phase shifter error of the *i*-th phase shifter, denoted by $\delta_{i}$, can be modeled as a uniformly-distributed random variable in the interval $\left[-\delta_{max},\delta_{max}\right]$ [27]:

(6) $$\delta_{i}\in \left[-\delta_{max},\delta_{max}\right],0\leq \delta_{max}\leq 180^{\circ },$$

where $\delta_{max}$ is a parameter depending on the phase shifter implementation [14,15] and the $\delta_{i}$ variables are assumed independent among different phase shifters. Therefore, if we define $\theta_{i}$ as the ideal phase shift to be applied by phase shifter *i*, the actual phase shift applied by that phase shifter will be $\theta_{i}^{\prime }=\theta_{i}+\delta_{i}$.

This work focuses on phase shifter errors constant over the bandwidth of interest but variable with time. Note that phase shifter errors that are constant with time are mainly caused by manufacturing imperfections, and, thus, they could be estimated in the uplink and their effect could be compensated in the beamforming stage. Otherwise, phase shifter errors variable with time cause a random and unpredictable error that cannot be estimated and compensated. The reason for selecting the latter assumption is to emulate the effect of using different beams for the channel estimation and transmission phases due to the inclusion of different phase-shifter errors in each of them. This may happen in frequency division duplex (FDD) systems. As a result, when the phase-shifter error varies over time, the beam selected from the codebook during the scheduling phase can result in a suboptimum beam for the transmission phase. Note that this is a worst-case scenario for the evaluation of the impact of phase-shifter errors.

Table 4 collects several performance results for an ABF scheme with P=4 affected by phase shifter errors, for three different values of the $\delta_{max}$ parameter in degrees: $\delta_{max}=2^{\circ },6^{\circ }$ and $10^{\circ }$. Note that, according to previous works [14,15], the value of $\delta_{max}=10^{\circ }$ is very unlikely in practice, and, thus, it is here included for a worst-case evaluation. In particular, average and 5th percentile user throughput values have been obtained considering only the UEs in LoS, only the UEs in NLoS, or both types combined. Average cell throughput values are also included. In all cases, the performance loss with respect to the ideal case is also shown. The results reveal that the impact of phase shifter errors is very minor, and it only causes a maximum reduction of 3.5% in user throughput, particularly for LoS users with a pessimistic value of $\delta_{max}=10^{\circ }$. Indeed, LoS UEs are more affected by this impairment because they strongly depend on the accuracy of the direct beam. In addition, since the equivalent receive beams of LoS UEs are narrower (in meters) than those of NLoS UEs, generally located farther away from the BS, an error in the pointing direction is more critical when serving LoS UEs.

### 5.2. Performance with Combiner Losses

In the analog beamforming scheme, the signals of the different RF chains are mixed together by means of a combiner to feed the antenna array. Non-ideal combiners are known to introduce a power loss in their outputs [28], which increases with the number of branches that the device has to combine. In this work, we denote with *L* the power loss of the basic combiner with two input branches, i.e., whose output power is given by the sum of the two input powers divided by *L*. Then, we assume that a combiner for a generic number *P* of RF chains is implemented by means of a cascade of log${}_{2}P$ two-branch combiners, leading to a total loss, expressed in logarithmic units:

(7) $$L^{\left(tot\right)}\left[dB\right]=\log_{2}P\cdot L\left[dB\right].$$

Note that, differently from the results with ideal hardware shown in Figure 3, where user throughput increases with the number of RF chains, by taking into account now the combiner power loss (7), there will be an optimum number of RF chains depending on the scenario. Indeed, when we increase the number of RF chains, on one side, we still increase the number of users that can be served by the BS (thus allowing higher spatial multiplexing), but, on the other side, we have a power loss that decreases the SINR measured at the UEs.

Regarding the selection of *L* values, note that the case without any compensation of losses corresponds to L=3 dB [16], where half of the input power is lost. However, in those cases where compensation of the losses is possible by adding power amplifiers after the combiner stages, realistic values for combiner losses range from 0 to 3 dB. In this sense, we selected L=1 dB as a meaningful intermediate evaluation point. It is worth noting that compensation of losses involves an additional cost due to including extra per-antenna power amplifiers.

**Figure 10:**
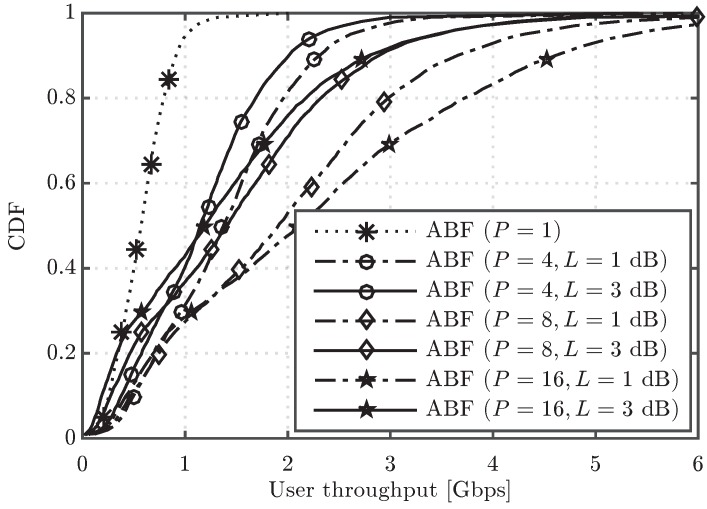
CDF of user throughput for the ABF scheme with different values of *P* and *L*.

Figure 10 shows the CDF of user throughput for ABF with different number of RF chains, P=4,8 and 16, and two values of the two-branch combiner loss parameter, L=1,3 dB. Results show that, for L=1 dB, the best results are obtained with P=16 RF chains. However, when L=3 dB is considered, the scheme with P=8 RF chains achieves the best performance. Therefore, the optimum number of RF chains depends strongly on the combiner losses.

The results in Figure 10 are complemented by Table 5, which collects the average UE throughput, 5th percentile UE throughput and average cell throughput for the same schemes. More specifically, the table contains the average values of each performance indicator in Gbps and also the performance loss with respect to having ideal combiners (L=0 dB). It is again observed that, for L=1 dB, P=16 RF chains is the best option, despite the fact that the higher the number of RF chains, the higher the total losses due to realistic combiners (for instance, the loss in average UE throughput is around 5% for P=4 but around 9% for P=16). However, for the P=16 case, the gain in average cell throughput is reduced from 10% with L=1 dB to more than 45% with L=3 dB. Furthermore, in the latter case, the degradation for the users in the cell edge is of around a factor of 4, which reinforces the conclusion that no more than eight parallel RF chains are recommended in the ABF scheme when L=3 dB. All the results show the great effect that the combiner losses have on the performance of the algorithms, especially when the number of RF chains gets higher.

Finally, it is worth noting that the optimum number of RF chains is different for the users in LoS and NLoS conditions. In fact, for L=1 dB, better results are obtained for both types of users when increasing the number of RF chains, as the user multiplexing offered by the massive MIMO setup compensates the degradation due to combining losses. On the other hand, for L=3 dB, LoS users achieve the best performance with eight RF chains, while NLoS users get higher throughputs when using only four RF chains. In the end, the percentage of LoS/NLoS users in the system together with the combiner losses will determine the optimum number of active RF chains for ABF.

## 6. Conclusions

This paper has presented a thorough performance evaluation of digital precoding and analog beamforming schemes in a massive MIMO multi-cell deployment operating in the mmW frequency band. In the first part, a comparison among analog and digital precoding schemes has been carried out under ideal assumptions, namely, perfect and timely CSI at the transmitters, ideal phase-shifters and combiners in the analog scheme and lack of PAPC in digital precoding. MU schemes have been compared against ABF without spatial multiplexing of users, considered here as the baseline scheme. It was observed that by scheduling multiple users at a time, the throughput is substantially increased. Simulation results have further shown that analog beamforming schemes can reach the performance of fully digital precoders when working under LoS conditions and with a sufficient number of parallel RF chains. On the other hand, analog schemes have been shown to be more sensitive to interference and, thus, more degraded in NLoS conditions, in which increasing the number of RF chains can not provide any advantage.

After the initial performance comparison, the effect of several realistic impairments has been included in the simulation setup. To this aim, the effect of having only outdated CSI for precoding at the BS has been firstly analyzed by considering a more realistic period to update the channel in all the evaluated schemes. In addition, PAPC have been set in the two considered digital precoding variants to also see the effect of this realistic constraint. Lastly, the errors introduced by both the phase shifters and combiners in the analog architecture have been modeled.

From the results with outdated channel information, it can be concluded that digital precoding schemes are more sensitive to imperfections on the CSI knowledge at the BS, presenting large losses (up to 34%) when the CSI update period is increased. On the other hand, analog beamforming schemes have shown to be robust against the channel information inaccuracy, presenting a negligible reduction of performance when the channel reporting period is increased.

The results with PAPC have exhibited a performance reduction in digital precoding schemes based on MRT of about 4%, which leads to a small reduction of the performance gap between digital and analog implementations. In practice, the performance loss due to the PAPC could be compensated, for instance, by means of a more elaborated scheduler with a more frequent update of precoders.

Regarding the effect of phase-shifter errors, simulation results have shown that this impairment has little impact on the ABF performance, even when the magnitude of the introduced phase error is significantly higher than current state-of-the-art values known from manufacturing. Nevertheless, LoS UEs suffer a stronger degradation due to this impairment when compared to the NLoS UEs, due to their narrower received beams. Conversely, combiner losses have indeed a great impact on the system performance, mainly when the number of RF chains in the analog scheme is high. In fact, the magnitude of the power loss introduced by the combiner determines in the end the optimum number of RF chains to be selected for analog beamforming. Overall, it is more suitable to employ a low number of RF chains when the losses introduced by the combiner are large.

Future work includes studying the performance of mmW beamforming methods with different planar array solutions and also the evaluation of more advanced hybrid precoding designs with an optimized baseband processing stage. In addition, analyzing the performance in the presence of more realistic user mobility would be of future interest.

## Figures and Tables

**Table 1:** Simulation parameters.

**Simulation time per drop**	1 s
**Number of drops**	10
**Carrier frequency**	28 GHz
**Number of FBs**	100
**FB bandwidth**	10 MHz
**Subframe duration**	1 ms
**Scheduling policy**	Proportional fair
**Thermal noise power spectral density**	−174 dBm/Hz
**Site transmit power**	40 dBm
**BS antenna tilt**	$12^{{}^{\circ}}$
**BS height**	10 m
**BS antenna element gain**	8 dBi
**BS antenna element front-to-back ratio**	20 dB
**BS antenna element half-power beamwidth**	$70^{{}^{\circ}}$ (Both H and V planes)
**UE noise figure**	7 dB
**UE speed**	3 km/h
**UE antenna pattern gain**	12 dBi
**UE antenna pattern front-to-back ratio**	20 dB
**UE antenna pattern half-power beamwidth**	$45^{{}^{\circ}}$ (Both H and V planes)
**UE height**	1.5 m
**Min UE-BS distance**	10 m

**Table 2:** Average number of different multiplexed users per subframe.

ABF (P=4)	ABF (P=8)	ABF (P=16)	DP NFS	DP FS
3.9	7.9	11.9	14.1	16.7

**Table 3:** Performance results with ideal assumptions.

	Avg UE Thr.		5%-ile UE Thr.		Avg Cell Thr.	
	Thr.	Gain	Thr.	Gain	Thr.	Gain
	[Gbps]	[%]	[Gbps]	[%]	[Gbps]	[%]
**ABF (P=1)**	0.59	-	0.20	-	9.8	-
**ABF (P=4)**	1.52	157	0.42	112	25.0	155
**ABF (P=8)**	2.22	276	0.42	111	36.4	272
**ABF (P=16)**	2.62	344	0.41	104	43.1	340
**DP NFS**	2.86	384	1.04	424	47.4	384
**DP FS**	2.96	401	1.10	453	49.0	400

**Table 4:** Performance results of the ABF scheme with P=4 including phase shifter errors.

	$\delta_{\max}=2^{{}^{\circ}}$		$\delta_{\max}=6^{{}^{\circ}}$		$\delta_{\max}=10^{{}^{\circ}}$	
	Thr.	Loss	Thr.	Loss	Thr.	Loss
	[Gbps]	[%]	[Gbps]	[%]	[Gbps]	[%]
**Avg UE thr. LoS**	1.871	0.12	1.847	1.41	1.807	3.55
**Avg UE thr. NLoS**	0.794	−0.03	0.785	1.10	0.770	2.94
**Avg UE thr.**	1.518	0.12	1.500	1.34	1.467	3.51
**5%-ile UE thr. LoS**	1.029	0.34	1.021	1.15	1.004	2.77
**5%-ile UE thr. NLoS**	0.311	−0.12	0.309	0.43	0.304	2.11
**5%-ile UE thr.**	0.419	0.51	0.415	1.44	0.410	2.62
**Avg cell thr.**	25.01	0.12	24.7	1.37	24.2	3.46

**Table 5:** Performance results of the ABF scheme with combiner losses for different numbers of RF chains.

	P=4				P=8				P=16			
	L=1 dB		L=3 dB		L=1 dB		L=3 dB		L=1 dB		L=3 dB	
	Thr.	Loss	Thr.	Loss	Thr.	Loss	Thr.	Loss	Thr.	Loss	Thr.	Loss
	[Gbps]	[%]	[Gbps]	[%]	[Gbps]	[%]	[Gbps]	[%]	[Gbps]	[%]	[Gbps]	[%]
**Avg UE thr. LoS**	1.775	5.25	1.518	18.99	2.693	6.15	2.082	27.43	3.180	8.45	1.941	44.12
**Avg UE thr. NLoS**	0.736	7.25	0.589	25.72	0.777	12.51	0.509	42.69	0.726	16.54	0.379	56.41
**Avg UE thr.**	1.436	5.52	1.215	20.10	2.067	6.89	1.574	29.08	2.377	9.23	1.438	45.11
**5%-ile UE thr. LoS**	0.963	6.76	0.782	24.29	1.256	7.21	0.860	36.40	1.264	8.39	0.683	50.53
**5%-ile UE thr. NLoS**	0.282	9.15	0.203	34.51	0.243	22.09	0.126	59.50	0.210	27.52	0.087	69.97
**5%-ile UE thr.**	0.382	9.37	0.281	33.38	0.340	18.66	0.178	57.51	0.300	25.85	0.119	70.53
**Avg cell thr.**	23.63	5.64	19.98	20.20	33.90	6.95	25.58	29.79	38.97	9.50	23.19	46.14
